# The ameliorative effects of choline on ethanol-induced cell death in the neural tube of susceptible BXD strains of mice

**DOI:** 10.3389/fnins.2023.1203597

**Published:** 2023-09-18

**Authors:** Fannia Xu, Jennifer D. Thomas, Dan Goldowitz, Kristin M. Hamre

**Affiliations:** ^1^University of British Columbia, Centre for Molecular Medicine and Therapeutics, Vancouver, BC, Canada; ^2^Center for Behavioral Teratology, San Diego State University, San Diego, CA, United States; ^3^Department Anatomy and Neurobiology, University Tennessee Health Science Center, Memphis, TN, United States

**Keywords:** recombinant inbred mice, fetal alcohol spectrum disorders, genetics, choline, apoptosis, forebrain, brainstem

## Abstract

**Introduction:**

Fetal alcohol spectrum disorders (FASD) are the leading preventable cause of intellectual disability, providing the impetus for evaluating various potential treatments to ameliorate ethanol’s teratogenic effects, particularly in the nervous system. One treatment is the dietary supplement choline which has been shown to mitigate at least some of ethanol’s teratogenic effects. The present study was designed to investigate the effects of genetics on choline’s efficacy in ameliorating cell death in the developing neural tube. Previously, we examined BXD recombinant inbred mice, and their parental C57BL/6 J (B6) and DBA/2 J strains, and identified strains that were sensitive to ethanol’s teratogenic actions. Thus, we used these strains to identify response to choline treatment.

**Materials and methods:**

Timed pregnant mice from 4 strains (B6, BXD51, BXD73, BXD2) were given either ethanol or isocaloric maltose-dextrin (5.8 g/kg in two administrations separated by 2 h) with choline at one of 3 doses: 0, 100 or 250 mg/kg. Subjects were exposed via intragastric gavage on embryonic day 9 and embryos were collected 7 h after the initial ethanol administrations. Cell death was analyzed using TUNEL staining in the developing forebrain and brainstem.

**Results:**

Choline ameliorated the ethanol-induced cell death across all 4 strains without causing enhanced cell death in control mice. Choline was effective in both the developing telencephalon and in the brainstem. Both doses diminished cell death, with some differences across strains and brain regions, although the 100 mg/kg dose was most consistent in mitigating ethanol-related cell death. Comparisons across strains showed that there was an effect of strain, particularly in the forebrain at the higher dose.

**Discussion:**

These results show that choline is effective in ameliorating ethanol-induced cell death at this early stage of nervous system development. However, there were some strain differences in its efficacy, especially at the high dose, providing further evidence of the importance of genetics in influencing the ability of choline to protect against prenatal alcohol exposure.

## Introduction

Fetal alcohol spectrum disorders constitute a constellation of phenotypes caused by exposure to ethanol during early development (e.g., Glass et al., 2023). Ethanol exposure has been shown to have teratogenic effects on the developing central nervous system (CNS), which can have life-long impacts for the exposed individual (Chokroborty-Hoque et al., 2014; Glass et al., 2023). Although the teratogenic effects of ethanol have been known for at least half a century and despite publicity campaigns, the incidence of FASD remains as high as 5 in every 100 live births (May et al., 2018, 2021). This has led to a focus on identifying treatments and therapeutics that can ameliorate ethanol’s teratogenic effects.

One of the most effective treatments, to date, has been choline supplementation. Choline is an essential nutrient that has several functions within the cell, influencing synthesis of phospholipids in cell membranes, synthesis of the neurotransmitter acetylcholine, and acting as a methyl donor in a number of cellular processes (Wallace et al., 2020). In animal models, choline has been shown to mitigate many, but not all, of the effects of developmental ethanol exposure during development ranging from brain to behavioral changes (Thomas et al., 2000, 2009, 2010; Monk et al., 2012; Bottom et al., 2020; Baker et al., 2022; Ernst et al., 2022; Naik et al., 2022). Choline is effective whether administered during prenatal alcohol or after birth (Thomas et al., 2009, 2010). Initial clinical studies have provided promising results that choline may be an effective treatment for at least some of ethanol’s teratogenic effects, particularly as they relate to the CNS (Jacobson et al., 2018; Wozniak et al., 2020; Warton et al., 2021; Ernst et al., 2022; Gimbel et al., 2022). In fact, choline deficiency has been shown to exacerbate ethanol’s teratogenic effects (Idrus et al., 2017).

Although choline continues to show promise as an effective intervention, it is critical to understand factors that can influence choline’s efficacy. One factor that has been proposed to be important is genetics. Two lines of evidence provide support for the role of genetics in choline’s efficacy. First, a study by Fischer and colleagues examined the relationship between the level of choline and its metabolite betaine in breast milk, and the amount of choline consumed in women with differing genetic backgrounds. Their results showed that polymorphisms in two choline-related enzymes, phosphatidylethanolamine-N-methyltransferase (PEMT) and methylenetetrahydrofolate dehydrogenase 1 (MTHFR), had a significant effect on either the basal levels of choline or on changes in the levels in breast milk following choline supplementation (Fischer et al., 2010a,b; Corbin and Zeisel, 2012). Second, a recent study examined children with FASD that were given choline supplementation or placebo and tested for tasks that assessed learning and memory functions along with concomitant evaluation of single nucleotide polymorphisms (SNPs) in various choline-metabolizing genes. The results demonstrated a significant association between SNPs in the gene for the choline transporter gene solute carrier family 44 member 1 (SLC44A1) and the ability to perform the tasks (Smith et al., 2021). The current study is designed to further evaluate the role of genetics in choline’s efficacy in ameliorating ethanol-induced cell death early in the development of the CNS.

Mouse models are frequently used to study the genetic pathways involved in FASD and possible candidates for the genetic variation. The BXD recombinant inbred (RI) panel is the largest mouse RI mapping panel, derived by crossing B6 and D2 mouse strains (Peirce et al., 2004). BXD recombinant inbred strains have often been used to study the genetics and epigenetics of alcoholism, since they show significant differences in their preference for ethanol (Cunningham, 1995; Philip et al., 2010). Additionally, a study by Downing et al. (2012) examined morphological phenotypes (such as abnormalities in the digits and kidney) across BXD strains following prenatal alcohol exposure and found differential sensitivity in these strains. Previous work in our lab identified strains of BXD mice that were more susceptible to ethanol’s induction of cell death in the neural tube (“high cell death strains”) and those that were less susceptible (“low cell death strains”). We examined three BXD strains that have been identified as high cell death strains in the present study: BXD2, BXD51, and BXD73 (although there was low cell death in the brainstem) (Theberge et al., 2019). Additionally, the parental B6 strain was also a high cell death strain and was thus also evaluated in the current study.

In the present study, we examined these 4 high cell death strains to address the following. (1) Previous work has shown that choline can ameliorate ethanol-induced cell death (Wang and Bieberich, 2010) and this study was undertaken to confirm the previous report *in vivo*. (2) Previous work in rats and sheep has suggested that, at certain doses and phenotypes examined, choline itself can be teratogenic (Goeke et al., 2018; Carugati et al., 2022) and this study was undertaken to determine if differing genetic backgrounds showed evidence of baseline teratogenicity due to choline. (3) Choline may improve performance on some, but not all, behavioral outcomes, so we examined two brain regions to assess choline’s efficacy across brain regions. (4) We evaluated two doses of choline, a moderate dose (100 mg/kg body weight) and a high dose (250 mg/kg body weight) to determine if one dose was more or less effective. (5) We evaluated the effects of the genetic background by determining if the effects were equivalent across embryos of differing genetic backgrounds.

## Materials and methods

### Animals

All animal work was conducted under the auspices of the Institutional Animal Care and Use Committee at the University of Tennessee Health Science Center. Previously, we determined the levels of ethanol-induced cell death in over 25 recombinant inbred BXD strains, as well as the parental C57BL/6 J (B6) and DBA/2 J mouse strains (Theberge et al., 2019). From this analysis we identified strains that were highly vulnerable to ethanol’s teratogenic insult and strains that were resistant to ethanol’s effects. In the present study we focused on the analysis of 4 of the most vulnerable strains: BXD2, BXD51, BXD73 and B6.

### Ethanol exposure, tissue collection and processing

The breeding and ethanol exposure paradigm was conducted as previously described (Theberge et al., 2019). Briefly, females were mated with males of the same genotype for 4 h beginning at 9 a.m. Vaginal plugs were checked and the presence of a plug denoted embryonic day zero (E0). On E9, each dam was weighed and given the appropriate solution via intragastric gavage. Because we previously showed little difference between non-handled and gavaged controls, only the gavaged controls were used and therefore, all dams received solutions via gavage. Dams were given either 5.8 g/kg of ethanol in sterile saline divided into 2 doses separated by 2 h or the control isocaloric solution of maltose/dextrin also in saline. Specific groups were also given choline chloride (Balchem Corp., New Hampton, NY, United States) at a dose of either 100 or 250 mg/kg. The choline was included in the solutions given via gavage to minimize the handling of the mice. In this study, the following 6 groups were included in each strain: (1) Maltose/dextrin no choline control group, (2) Maltose/dextrin +100 mg/kg choline, (3) Maltose/dextrin +250 mg/kg choline, (4) Ethanol no choline group, (5) Ethanol +100 mg/kg choline, (6) Ethanol +250 mg/kg choline. Seven hours after the first injection, dams were euthanized, the individual embryos were dissected out of the uterus, and embryos with closed anterior neuropores were immersion fixed in 4% paraformaldehyde in 0.1 M phosphate buffered saline (PBS) for 1 h (embryos with open anterior neuropores were discarded). The somite number at this point of development was typically between 15 and 25. After 1 h, the embryos were removed from the fixative and placed in PBS at 4°C. Embryos were subsequently embedded in paraffin using standard techniques. Single embryos were embedded for sectioning in the horizontal plane. Embryos were serially sectioned at 8 um and mounted on glass slides (Fisher Scientific).

### Detection of apoptotic cell death

Slides with sections of the developing telencephalon and brainstem were selected for staining to demonstrate dead and dying cells in the neuroepithelia. Terminal deoxynucleotidyl transferase dUTP nick end labeling (TUNEL) assays were used to label cells undergoing apoptotic DNA fragmentation (Nikolic et al., 2000). TUNEL-positive cells were labelled using the ApopTag *In Situ* Apoptosis Kit (Millipore Sigma) according to the manufacturer’s protocol. Sections were deparaffinized and rehydrated with graded ethanols, incubated with 20 μg/mL of proteinase K, blocked with 2% hydrogen peroxide, and incubated with terminal deoxynucleotidyl transferase (TdT) followed by an anti-digoxigenin peroxidase conjugated antibody. 3,3′-diaminobenzidine (DAB) was used to visualize the digoxigenin-labelled DNA fragments. Sections were counterstained with 0.5% methyl green to provide contrast with the DAB staining. Labelled TUNEL-positive (apoptotic) cells are brown, and non-apoptotic cells are green. The sections were dehydrated and coverslipped with Permount (Fisher Scientific).

### Quantification of cell death

TUNEL-positive cells were manually counted within the regions of interest using a brightfield microscope with a 40x objective (Standard Zeiss ICS Brightfield Microscope). Cells undergoing apoptosis were defined as cells with brown TUNEL staining within the confines of the cell, or tightly clustered brown TUNEL staining. Cells with only a faint brown stain that was not confined within the cell were not counted as TUNEL-positive.

The telencephalon and brainstem were the regions of interest for the determination of cell death. The telencephalon was demarcated as the neuroepithelium rostral to the optic vesicles; the brainstem was demarcated as the neuroepithelium at the level of the otic placodes (Supplementary Figure S1). For each region of interest, 2 sections per region were sampled per embryo, and 3 embryos were quantified per litter. The first sections analyzed in the forebrain and hindbrain were at the level of the optic and otic vesicles, respectively. The second sections for analysis were at least 32 um away to ensure that the same cell was not counted twice. For each section, the whole brainstem or forebrain was analyzed.

To quantify the area of the region of interest, a Zeiss 200 M Axiovert Inverted Microscope with AxioVision 4.6 software (Carl Zeiss Microscopy, Jena, Germany) was used to image the tissue. Images of brainstem and forebrain sections were taken using a 20x objective at preset dimensions of 1,300 × 1,030 pixels. Images taken from the Zeiss Inverted Microscope were uploaded into the software ImageJ version 1.53c (National Institutes of Health, Bethesda, MD), which was used to calculate the area of the regions of interest. The region of interest was selected using the freehand selection tool to measure the area. The image was scaled at 1,900 pixels per mm to convert the area measured in square pixels to equivalent dimensions in mm^2^.

The average density of apoptotic cells in the brainstem and forebrain was calculated by dividing the number of TUNEL-positive cells in each region of interest by the area of the region (in mm^2^) for each sampled section. For each embryo, the average TUNEL-positive cells per mm^2^ for all sections sampled in a given region from that embryo yielded an apoptotic cell density for that region. Finally, an average value for TUNEL-positive cells per mm^2^ for each embryo in a litter was used to calculate the average apoptotic cell density for that litter for each brain region. The litter means for cell death were used as the unit of analysis. For each strain, 5–8 litters were analyzed for each EtOH and choline dose, and 3–5 litters were analyzed for each MD and choline dose.

### Data analysis

Three levels of analysis were applied to the data using linear regression models with significance set at *p* < 0.05 using IBM SPSS Statistics Version 29.0. The first level of analysis examined each brain region across all strains and doses to assess the efficacy of choline in ameliorating apoptotic cell death. The second level of analysis compared whether there were dose or regional differences within each strain. The third level of analysis was conducted to compare choline’s ability to reduce cell death toward control values across the different strains. Because there were differences in baseline levels of ethanol-only induced cell death across strains, we calculated adjusted values to compare data between strains. The adjusted value was determined by subtracting the average cell death of the MD-treated controls that were not administered choline from each individual litter cell death average of the ethanol+choline treatment groups. Any adjusted cell death values that were negative were set at zero.

## Results

### Choline is effective in ameliorating ethanol-induced cell death

**Figure 1:**
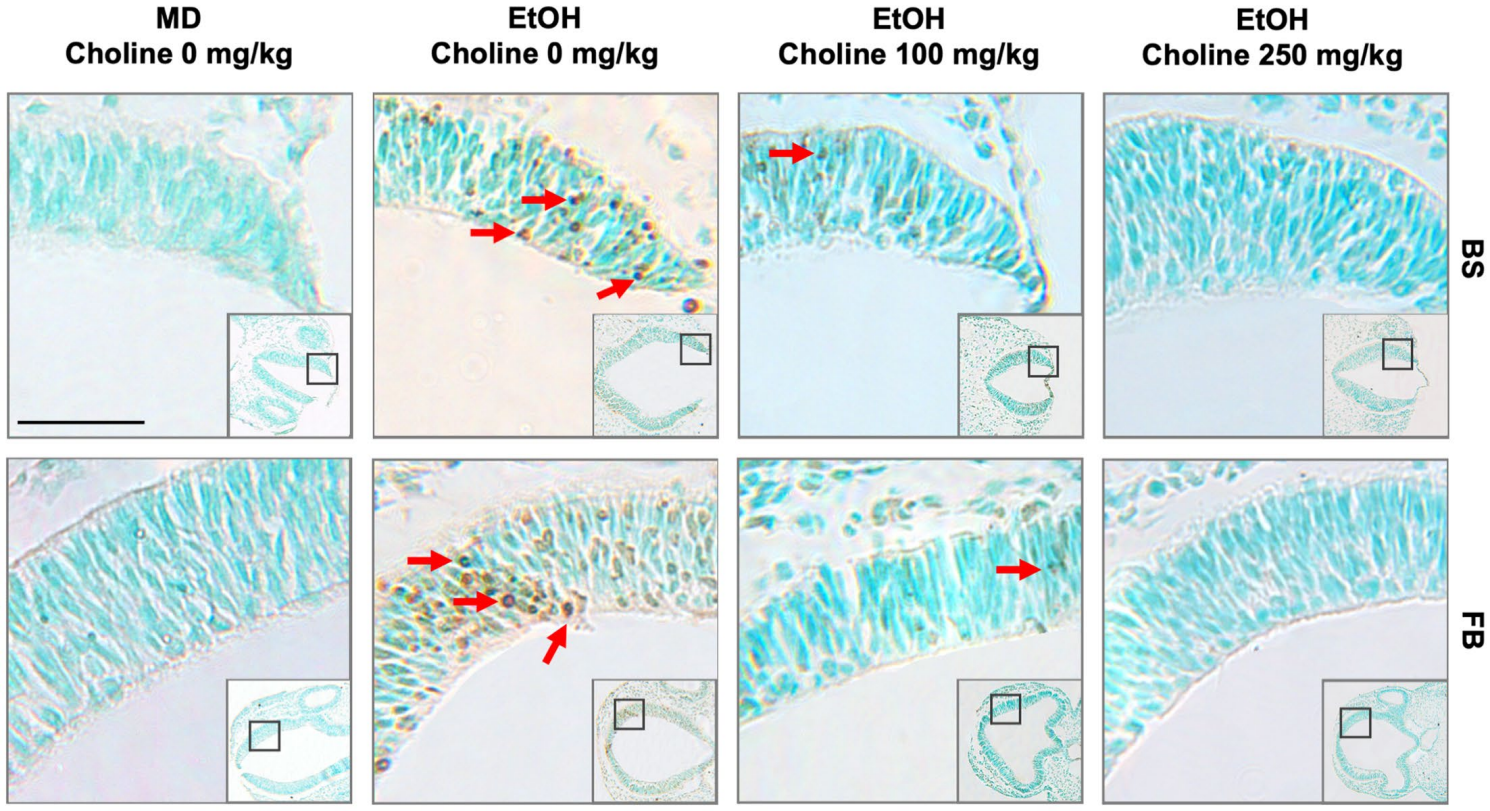
Choline is effective in ameliorating ethanol-induced cell death in the brainstem and forebrain as shown in the BXD51 mouse strain as illustrative of all 4 strains examined. Representative images of TUNEL-stained sections at E9.5 to illustrate apoptotic cells in each of the treatment conditions (top of panels) and brain regions (right of panels). TUNEL staining of apoptotic cells in brainstem (top, BS) and forebrain (bottom, FB) of BXD51 mice treated with MD or ethanol on embryonic day 9 (low magnification inset indicates the region of embryonic brainstem or forebrain shown at higher magnification). Treatment was supplemented by a choline dose of 0, 100, or 250 mg/kg. Dark deposits of DAB indicate cells undergoing apoptosis (examples highlighted by red arrows). Cells with lighter brown staining are associated with transit through the cell cycle and/or background and were not counted. Cells were counterstained with methyl green. MD = maltose-dextrin. Scale bars = 50 mm.

**Figure 2:**
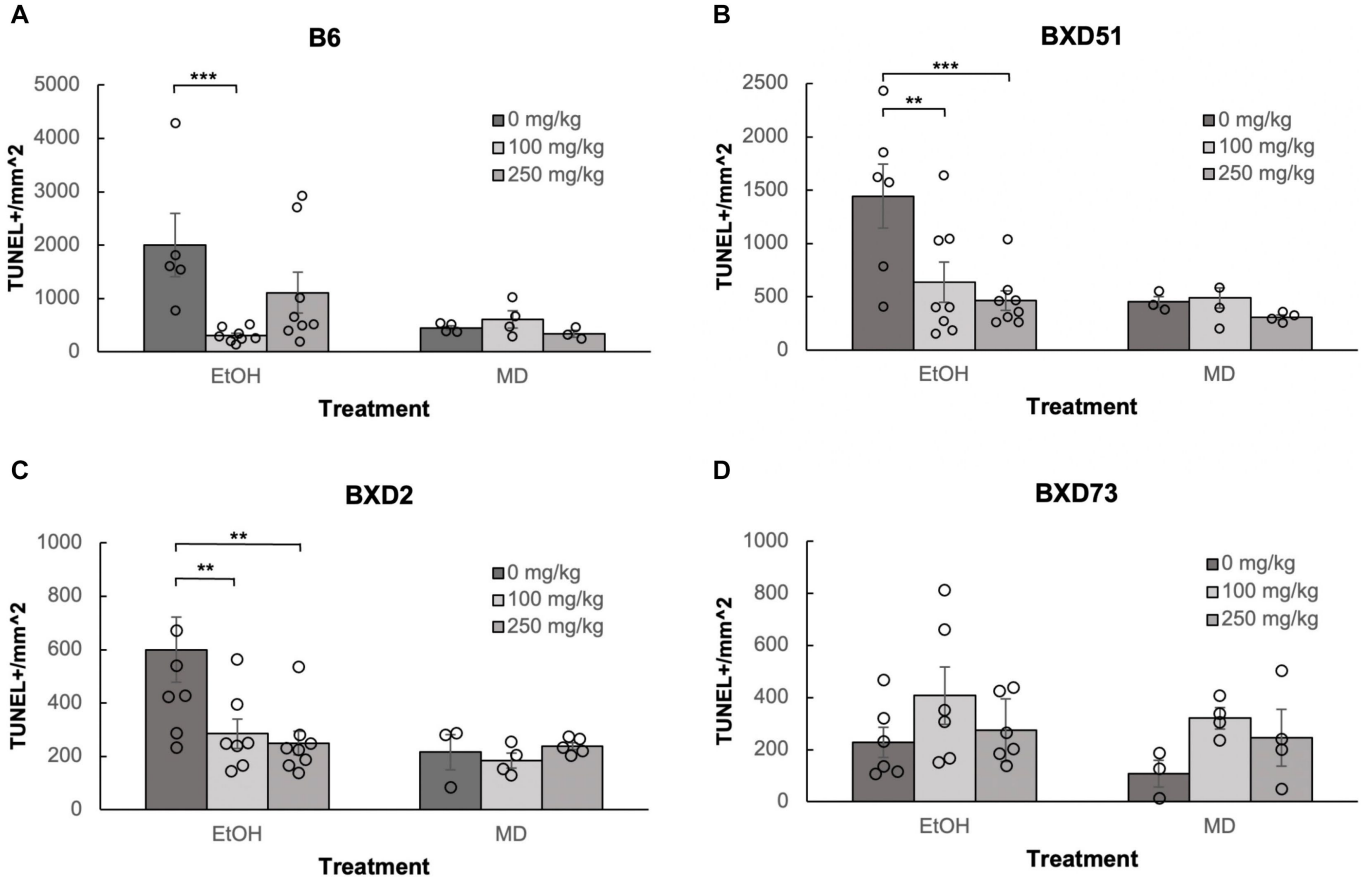
Choline is effective in ameliorating ethanol-induced cell death in the brainstem of B6, BXD51, and BXD2 embryos. Graphs represent the amount of cell death in the brainstem of ethanol-treated and maltose-dextrin (MD) control embryos that also received choline treatment with either 0 mg/kg, a moderate (100 mg/kg), or high (250 mg/kg) dose. Statistical comparisons among the three ethanol-treated groups are shown in the graphs. Choline+EtOH and choline+MD treated groups were compared at the same dose and there were no statistically significant differences. There were no statistically significant differences between the three MD-treated groups in any of the strains examined. **(A)** Significant difference in the level of cell death between embryos that do not receive choline and embryos that receive 100 mg/kg choline in the B6 strain. There is no significant effect of the 250 mg/kg dose on cell death. For EtOH-treated embryos, *N* = 5 for 0 mg/kg and *N* = 8 for 100 mg/kg and 250 mg/kg. For MD-treated embryos, *N* = 4 for 0 mg/kg and 100 mg/kg and *N* = 3 for 250 mg/kg. **(B)** Significant differences in levels of cell death between embryos that do not receive choline and embryos that receive 100 or 250 mg/kg choline in the BXD51 strain. For EtOH-treated embryos, *N* = 6 for 0 mg/kg and *N* = 8 for 100 mg/kg and 250 mg/kg. For MD-treated embryos, *N* = 3 for 0 mg/kg and 100 mg/kg and *N* = 4 for 250 mg/kg. **(C)** Significant differences in levels of cell death between embryos that do not receive choline and embryos that receive 100 or 250 mg/kg choline in the BXD2 strain. For EtOH-treated embryos, *N* = 8 for 0 mg/kg and 250 mg/kg and *N* = 7 for 100 mg/kg. For MD-treated embryos, *N* = 3 for 0 mg/kg, *N* = 4 for 100 mg/kg, and *N* = 5 for 250 mg/kg. **(D)** No significant differences are seen between embryos that do not receive choline treatment and embryos that receive either 100 or 250 mg/kg choline in the BXD73 strain. For EtOH-treated embryos, *N* = 6 for 0 mg/kg, 100 mg/kg and 250 mg/kg. For MD-treated embryos, *N* = 3 for 0 mg/kg and *N* = 4 for 100 mg/kg and 250 mg/kg. Error bars indicate standard error of the mean (SEM). ^*^p < 0.05, ^**^p < 0.01, ^***^p < 0.001.

**Figure 3:**
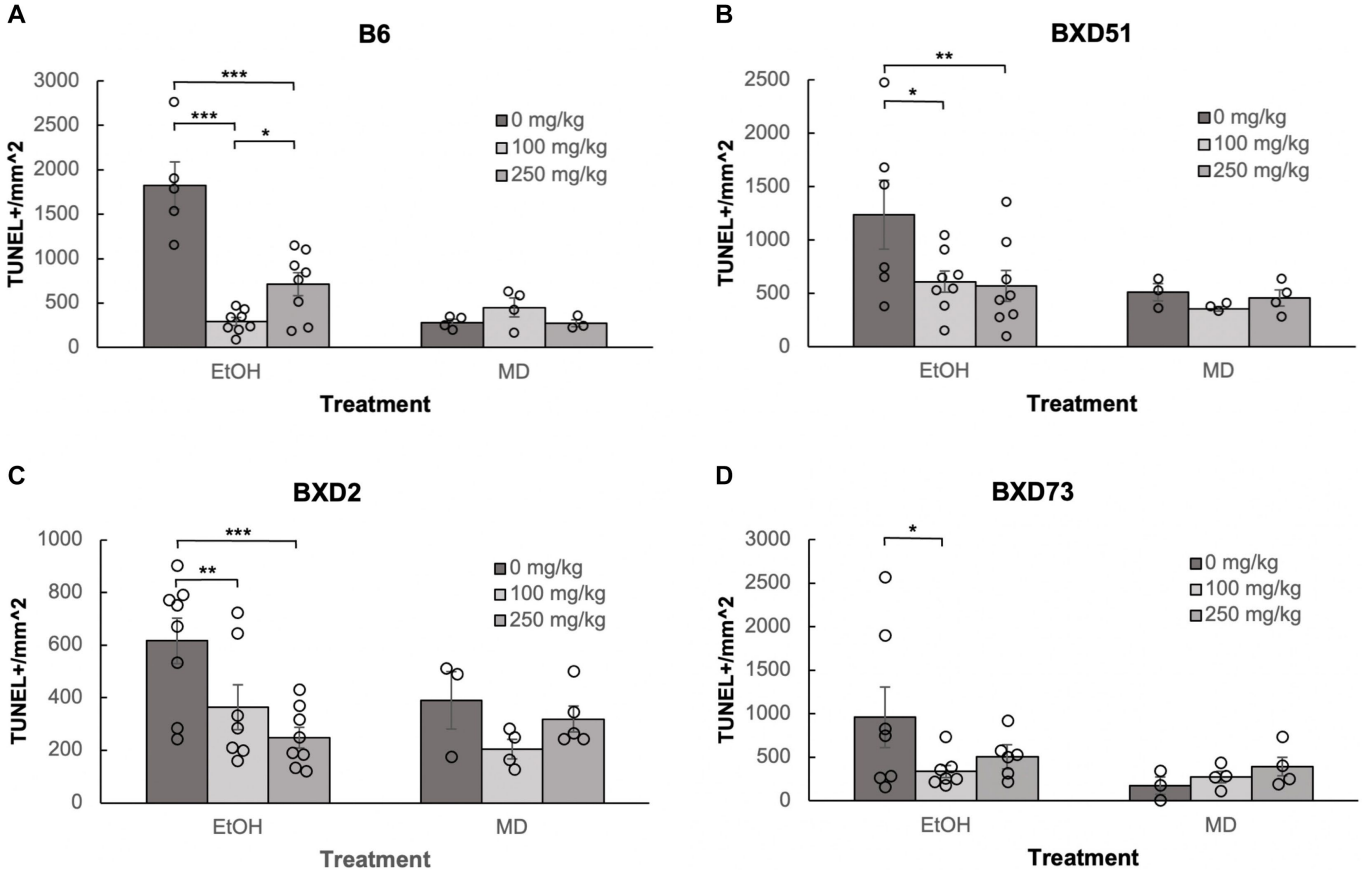
Choline is effective in ameliorating ethanol-induced cell death in the forebrain of B6, BXD51, BXD2, and BXD73 embryos. Graphs represent the amount of cell death in the forebrain of ethanol-treated and maltose-dextrin (MD) control embryos that also received choline treatment with either 0 mg/kg, a moderate (100 mg/kg) or high (250 mg/kg) dose of choline. Statistical comparisons among the three ethanol-treated groups are shown in the graphs. Choline+EtOH and choline+MD treated groups were compared at the same dose and there were no statistically significant differences. There were no statistically significant differences between the three MD-treated groups in any of the strains examined. **(A)** There are significant differences in levels of cell death between embryos that did not receive choline and embryos that received 100 or 250 mg/kg choline in the B6 strain. The 100 mg/kg dose was more effective in reducing cell death than the 250 mg/kg dose. For EtOH-treated embryos, *N* = 5 for 0 mg/kg, *N* = 8 for 100 mg/kg and 250 mg/kg. For MD-treated embryos, *N* = 4 for 0 mg/kg, 100 mg/kg and *N* = 3 for 250 mg/kg. **(B)** There are significant differences in levels of cell death between embryos that did not receive choline and embryos that received 100 or 250 mg/kg choline in the BXD51 strain. For EtOH-treated embryos, *N* = 6 for 0 mg/kg, *N* = 8 for 100 mg/kg and 250 mg/kg. For MD-treated embryos, *N* = 3 for 0 mg/kg, 100 mg/kg and *N* = 4 for 250 mg/kg. **(C)** There are significant differences in levels of cell death between embryos that did not receive choline and embryos that received 100 or 250 mg/kg choline in the BXD2 strain. For EtOH-treated embryos, *N* = 8 for 0 mg/kg, 250 mg/kg and *N* = 7 for 100 mg/kg. For MD-treated embryos, *N* = 3 for 0 mg/kg, *N* = 4 for 100 mg/kg, and *N* = 5 for 250 mg/kg. **(D)** There is a significant difference in the level of cell death between embryos that did not receive choline and embryos that received 100 mg/kg choline in the BXD73 strain. There was no significant effect of the 250 mg/kg dose on cell death. For EtOH-treated embryos, *N* = 6 for 0 mg/kg, *N* = 7 for 100 mg/kg and 250 mg/kg. For MD-treated embryos, *N* = 3 for 0 mg/kg, *N* = 4 for 100 mg/kg and 250 mg/kg. Error bars indicate standard error of the mean (SEM). ^*^p < 0.05, ^**^p < 0.01, ^***^p < 0.001.

One of the aims of our study was to confirm previous reports that choline can ameliorate ethanol-induced cell death. To test this, we quantified the levels of apoptosis in E9.5 embryos that were exposed to EtOH and administered either a moderate (100 mg/kg) or high (250 mg/kg) dose of choline and compared the amount of cell death to control embryos that were not administered choline. Choline was able to effectively reduce the amount of ethanol-induced cell death in the developing neural tube. In all 4 strains, there were fewer apoptotic cells and fewer clusters of apoptotic cells in the choline-treated embryos compared to controls that did not receive choline (Figure 1). Quantitative comparisons of apoptotic cell density showed that both the 100 mg/kg (*χ^2^* (1, *N* = 54) = 10.096, *p* = 0.001 for brainstem and *χ^2^* (1, *N =* 54) = 16.656, *p* < 0.001 for forebrain) and 250 mg/kg (*χ^2^* (1, *N* = 55) = 6.241, *p* = 0.012 for brainstem and *χ^2^* (1, *N =* 55) = 8.361, *p* = 0.004 for forebrain) doses of choline effectively reduced cell death in both regions of interest across all strains (Figures 2, 3; Supplementary Figure S2).

### Choline does not increase cell death in the developing neural tube

We administered choline to control, M/D treated litters to determine whether there were increases in cell death in the 4 strains of mice used in this study. Previous work has suggested that choline itself, at higher doses, may in fact be teratogenic (Goeke et al., 2018; Carugati et al., 2022). We administered choline to control, M/D treated litters to determine whether there increases in cell death in the 4 strains of mice used in this study. Patterns of apoptosis in both the 100 mg/kg and 250 mg/kg doses were similar to controls that did not receive any choline (Figure 1). Comparisons were done within each strain for each brain region. Within each strain, neither dose caused an increase in cell death (*p* > 0.05 for all strains and regions), and both doses had comparable levels of cell death to controls that were not administered choline (Figures 2, 3). While there were moderate differences between doses, levels of apoptosis did not reach comparable levels to ethanol-induced cell death. This shows that there are no baseline differences in choline-induced cell death in any of the strains examined.

### Choline was effective in reducing cell death at both dosages used

Neither dose was effective in the brainstem of BXD73 embryos (*χ^2^* (1, *N* = 12) = 3.176, *p* = 0.075 for 100 mg/kg and *χ^2^* (1, *N* = 12) = 0.209, *p* = 0.647 for 250 mg/kg). We also aimed to identify whether there are dose differences (100 vs. 250 mg/kg) in choline’s efficacy to reduce ethanol-induced cell death by comparing levels of cell death across different choline doses within each strain using a linear regression model. Both doses effectively reduced alcohol-related apoptosis in the brainstem of the BXD51 (*χ^2^* (1, *N =* 14) = 9.461, *p* = 0.002 for 100 mg/kg and *χ^2^* (1, *N =* 14) = 14.024, *p* < 0.001 for 250 mg/kg) and BXD2 (*χ^2^* (1, *N* = 15) = 7.933, *p* = 0.005 for 100 mg/kg and *χ^2^* (1, *N* = 16) = 10.515, *p* = 0.001 for 250 mg/kg) strains (Figure 2). Only the 100 mg/kg dose was effective in reducing apoptosis in the brainstem for the B6 strain (*χ^2^* (1, *N* = 13) = 12.085, *p* < 0.001) while the 250 mg/kg dose failed to significantly reduce apoptosis (*χ^2^* (1, *N* = 13) = 3.341, *p* = 0.068). Neither dose was effective in the brainstem of BXD73 embryos (*p* = 0.075 for 100 mg/kg and *p* = 0.647 for 250 mg/kg), which was not surprising given that the baseline level of ethanol-induced cell death was lower for this strain in the brainstem region. In the forebrain, both doses were effective in B6 (*χ^2^* (1, *N* = 13) = 61.261, *p* < 0.001 for 100 mg/kg and *χ^2^* (1, *N* = 13) = 32.276, *p* < 0.001 for 250 mg/kg), BXD51 (*χ^2^* (1, *N* = 14) = 6.146, *p* = 0.013 for 100 mg/kg and *χ^2^* (1, *N =* 14) = 6.941, *p* = 0.008 for 250 mg/kg), and BXD2 (*χ^2^* (1, *N* = 15) = 6.783, *p* = 0.009 for 100 mg/kg and *χ^2^* (1, *N =* 16) = 15.425, *p* < 0.001 for 250 mg/kg) strains, but only the 100 mg/kg dose (*χ^2^* (1, *N* = 14) = 4.823, *p* = 0.028) effectively reduced cell death in the BXD73 strain (*χ^2^* (1, *N* = 13) = 2.375, *p* = 0.123 for 250 mg/kg) (Figure 3). There were no significant differences in efficacy between the 100 and 250 mg/kg doses in any strain or region except in the forebrain of the B6 strain (*χ^2^* (1, *N* = 16) = 5.986, *p* = 0.014) where the 100 mg/kg dose was more effective in ameliorating ethanol-induced cell death.

Another way to view these data is to calculate the percent reduction by choline in ethanol-induced cell death (Table 1). This was obtained by dividing [(choline + ethanol) – (MD)] by [(Ethanol) – (MD)]. In the brainstem, the 100 mg/kg dose reduced cell death by 100% in the B6 strain, 81.1% in the BXD51 strain, and 81.9% in the BXD2 strain. The 250 mg/kg dose reduced cell death by 57.6% in the B6 strain, 98.8% in the BXD51 strain, and 91.1% in the BXD2 strain. In the forebrain, the 100 mg/kg dose reduced cell death by 99.4% in the B6 strain, 86.3% in the BXD51 strain, 100% in the BXD2 strain, and 79.1% in the BXD73 strain. The 250 mg/kg dose reduced cell death by 72.2% in the B6 strain, 91.7% in the BXD51 strain, 100% in the BXD2 strain, and 57.7% in the BXD73 strain. There were no statistically significant differences between the ethanol+choline group and the MD control group that did not receive choline.

**Table 1:** Percentage reduction in ethanol-induced cell death following choline treatment in the brainstem and forebrain across strains. The values were calculated as described in the Results.

	BRAINSTEM		FOREBRAIN	
Choline Dose	100 mg/kg	250 mg/kg	100 mg/kg	250 mg/kg
B6	100%	57.6%	99.4%	72.2%
BXD51	81.1%	98.8%	86.3%	91.7%
BXD2	81.9%	91.1%	100%	100%
BXD73	0%^*^	0%^*^	79.1%	57.7%

^*^ There was no percentage reduction in cell death in the brainstem of BXD73 embryos because ethanol causes little cell death in the brainstem of this strain (see Discussion).

### There were limited differences between forebrain and brainstem in the effectiveness of choline

We compared cell death in the developing brainstem and telencephalon to assess whether choline is similarly effective across different brain regions. Overall, choline was able to reduce cell death in both the brainstem and forebrain. There were no significant regional differences in choline efficacy in 3 of the 4 strains we examined. In the BXD73 strain, there was a significant regional difference in choline’s ability to reduce cell death at the 250 mg/kg dose (*χ^2^ (1, N = 12)* = 5.173, *p* = 0.023). Choline was effective in reducing apoptosis in the forebrain but not brainstem of BXD73 embryos.

### There were choline dose-dependent strain differences in efficacy

**Figure 4:**
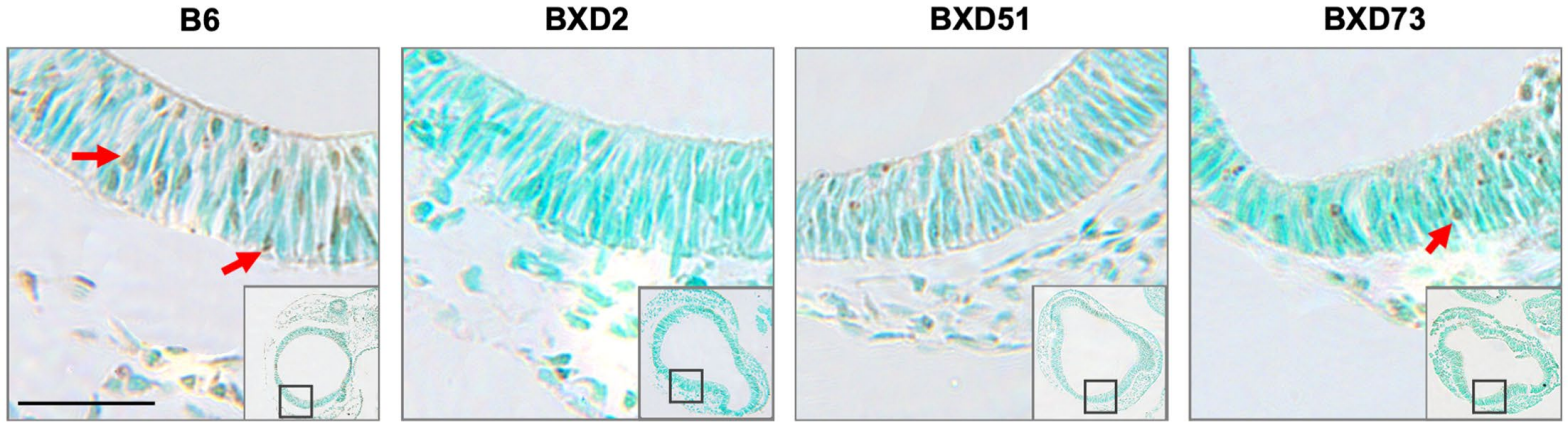
Strain differences in levels of TUNEL-positive cells in the forebrain across mouse strains exposed to ethanol and 250 mg/kg choline. Representative images of TUNEL-stained sections at E9.5 to illustrate apoptotic cells in B6, BXD2, BXD51, and BXD73 mouse forebrain (low magnification inset indicates the region of embryonic forebrain shown at higher magnification). Dark deposits of DAB indicate cells undergoing apoptosis (examples highlighted by red arrows). Cells were counterstained with methyl green. Scale bars = 50 mm.

**Figure 5:**
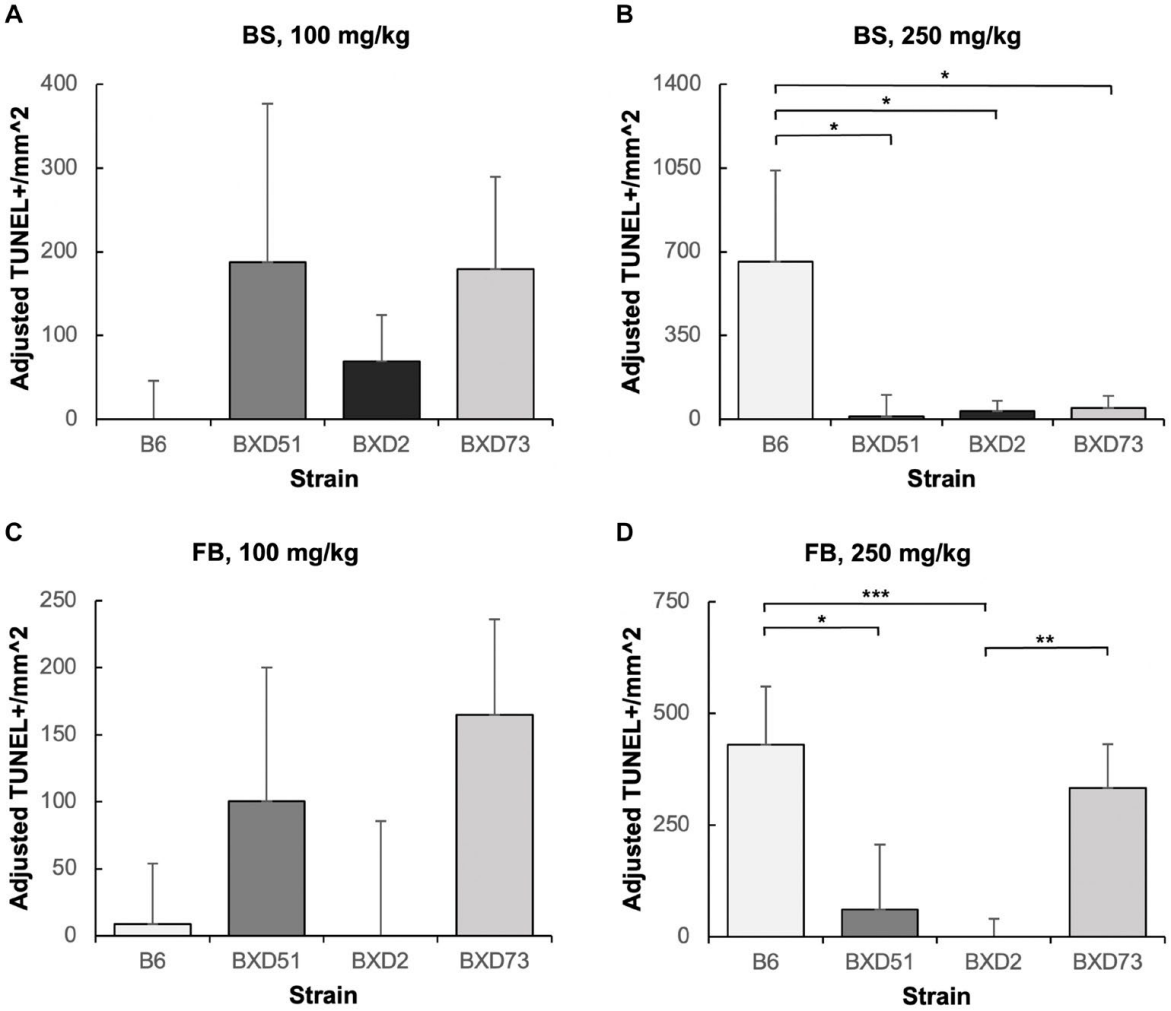
Strain differences in choline’s ability to ameliorate ethanol-induced cell death. Graphs represent adjusted values for cell death in the developing brainstem (BS) and forebrain (FB) after administration of moderate (100 mg/kg) and high (250 mg/kg) doses of choline. Adjusted TUNEL+ cells/mm^2^ values were calculated by subtracting the average cell death of the MD-treated controls that were not administered choline from each individual litter cell death average of the ethanol+choline treatment groups. Any adjusted cell death values that were negative were set at zero. It should be noted that there are substantial differences in the y-axis scales between panels as well as between the above graphs and those in Figures 2, 3. **(A)** No strain differences were seen in the brainstem (BS) region of ethanol-treated embryos administered a 100 mg/kg dose of choline. *N* = 8 for B6 and BXD51, *N* = 7 for BXD2, and *N* = 6 for BXD73. **(B)** There are significant differences between the B6 strain and all 3 of the BXD strains in the forebrain (FB) of ethanol-treated embryos administered a 250 mg/kg dose of choline. *N* = 8 for B6, BXD51, and BXD2, and *N* = 6 for BXD73. **(C)** No strain differences were seen in the forebrain (FB) region of ethanol-treated embryos administered a 100 mg/kg dose of choline. *N* = 8 for B6 and BXD51, and *N* = 7 for BXD2 and BXD73. **(D)** There are significant differences between the B6 strain and both the BXD51 and BXD2 strains as well as between the BXD2 and BXD73 strains in the forebrain (FB) of ethanol-treated embryos administered a 250 mg/kg dose of choline. *N* = 8 for B6, BXD51, and BXD2, and *N* = 7 for BXD73. Error bars indicate standard error of the mean (SEM). ^*^p < 0.05, ^**^p < 0.01, ^***^p < 0.001. Note the differences in the y-axis scales between panels.

One key aim of this study was to determine whether there are genetic differences in choline’s efficacy in ameliorating ethanol-induced cell death. Previous research shows that there are genetic differences in susceptibility to ethanol’s teratogenic effects (Theberge et al., 2019). Thus, we suspected that there may also be genetic differences in response to choline treatment. To test this, we compared the effectiveness of choline to reduce ethanol-induced cell death to levels seen in MD controls. We assessed levels of apoptosis in 4 different strains in mice that were either administered 100 mg/kg, 250 mg/kg, or no choline using the adjusted cell death values as defined in the Materials and Methods section. Overall, there are strain differences in choline’s efficacy in reducing ethanol-induced cell death (Supplementary Figure S3). Qualitative assessment of treatment with a 250 mg/kg dose of choline shows differences in the patterns of cell death in the developing forebrain across different strains of mice (Figure 4). Quantitative assessment of cell death and statistical analysis show that there are strain differences (Figure 5). There were significant differences across all 4 strains in both the brainstem (*χ^2^* (3, *N* = 30) = 7.908, *p* = 0.048) and forebrain (*χ^2^* (3, *N =* 30) = 18.609, *p* < 0.001) of embryos administered the 250 mg/kg dose of choline. When comparing between individual strains, significant differences were found between the B6 strain and all 3 of the BXD strains in the brainstem at the 250 mg/kg dose (*χ^2^* (1, *N* = 16) = 5.680, *p* = 0.017 for BXD51, *χ^2^* (1, *N* = 16) = 5.302, *p* = 0.021 for BXD2, and *χ^2^* (1, *N =* 14) = 4.373, *p* = 0.037 for BXD73). There were also significant differences between the B6 and both the BXD51 (*χ^2^* (1, *N =* 16) = 6.444, *p* = 0.011) and BXD2 (*χ^2^* (1, *N* = 16) = 15.513, *p* < 0.001) strains as well as between the BXD2 and BXD73 strains (*χ^2^* (1, *N* = 14) = 9.162, *p* = 0.002) in the forebrain at the 250 mg/kg dose. There was no significant effect of strain at the 100 mg/kg dose in either region of interest (*χ^2^* (3, *N* = 29) = 6.178, *p* = 0.103 for brainstem and *χ^2^* (1, *N =* 30) = 4.119, *p* = 0.249 for forebrain).

## Discussion

The present study demonstrates that choline was effective in ameliorating ethanol-induced cell death in the early developing neural tube. This is consistent with the study by Wang and Bieberich (2010) that demonstrated that choline treatment prevented ethanol-induced cell death at the same age in neural crest derived cell cultures. The ability of choline to reduce apoptosis in the face of other insults, such as folate deficiency, has also been shown at older ages (Craciunescu et al., 2003), suggesting that choline may protect against cell death at multiple developmental periods. This study provides additional validation for the use of choline as a potential treatment for ethanol-induced teratogenesis and demonstrates that even in early prenatal development of the CNS, choline can mitigate ethanol-related neuropathology.

It is interesting to note that both doses of choline were shown to be effective in ameliorating the cell death induced by ethanol (see Figures 2, 3). In the present study, both 100 mg/kg and 250 mg/kg doses of choline were used, and both of these doses have been used in previous studies and have been shown to be effective in different paradigms in ameliorating a range of phenotypes that are both behavioral and morphological (Thomas et al., 2000, 2009, 2010; Monk et al., 2012; Baker et al., 2022; Ernst et al., 2022; Naik et al., 2022). However, it is interesting to note that the low dose of choline was more consistent than the high dose in ameliorating ethanol-induced cell death, providing a potential cautionary warning about high doses.

Equally as important, these results show little evidence for enhanced ethanol-induced cell death caused by choline treatment. This is shown both by the lack of effect of choline in the control animals and the lack of additional cell death in the ethanol-exposed embryos that also received choline. Our findings align with the majority of previous studies which have shown that choline is safe for the developing embryo (Thomas et al., 2009, 2010; Kwan et al., 2021; Baker et al., 2022) although there have been a few exceptions, particularly at high doses such as 100 mM (Goeke et al., 2018; Carugati et al., 2022).

One of the important findings of this study is that there are strain differences in choline’s ability to ameliorate ethanol-induced cell death but only at the higher dose. This adds to the growing literature that suggests that genetics play an important role in choline’s efficacy (e.g., Smith et al., 2021). This is consistent with previous studies showing that allelic differences in various enzymes in the choline metabolic pathway can impact its efficacy (Fischer et al., 2010a,b). Further research is needed to assess whether these allelic differences are present within these BXD RI strains. Moreover, these results suggest that, at low doses, the effect of genetics is less apparent which could have important implications for its use in human populations.

These studies show the efficacy of choline but do not provide clues as to the mechanism of choline’s protection. Choline has numerous effects but three are most likely to act within the CNS. First, choline is part of the pathway for synthesis of the neurotransmitter acetylcholine (ACh). At older ages, it is likely that this is a relevant mechanism for choline’s efficacy. However, at this early developmental stage, it is unclear how much ACh is present (Schambra et al., 1989; Abreu-Villaca et al., 2011) and therefore this mechanism is unlikely to be important at this age. Second, it is a component of phospholipid synthesis for cell membrane components. Ethanol can affect membrane composition (e.g., Naik and Ramadoss, 2023) and while it is unclear whether this is the mechanism of choline’s efficacy, it is a strong potential mechanism. Third, it is a methyl donor that is essential for methylation which is an important epigenetic modification. Several studies have shown that ethanol exposure during development can alter DNA methylation and choline can modify ethanol’s effects (Balaraman et al., 2017; Sarkar et al., 2019). Previously, we examined several epigenetic modifications across multiple brain regions in neonatal mice given ethanol 7 h prior to tissue collection (Schaffner et al., 2020). The results demonstrated that there were both altered DNA methylation and altered gene expression in this short timeframe, suggesting that altered methylation of either DNA or histones could be a significant mechanism for choline’s protective actions with the early neural tube.

Our results indicate that whatever the mechanism(s) of neural protection is/are, it needs to help explain the complex nature of the protective effects: the strain-, dose-, and region-specific differences seem amongst the four strains that were analyzed in this study. Data from human clinical trial work using choline has discovered allelic differences in key choline utilization genes, cellular choline transporter gene solute carrier family 44 member 1 (SLC44A1) (Smith et al., 2021), methylenetetrahydrofolate dehydrogenase 1 (MTHFR, also known as Mthfd1 in the mouse) (Fischer et al., 2010a) and phosphatidylethanolamine-N-methyltransferase (PEMT) (Fischer et al., 2010a,b) that alter choline related phenotypes. To speak to the polymorphisms found in humans using a mouse model, it would be interesting to examine SNPs in reference populations like the BXD RI lines to see if a gene of interest can have a phenotypic impact. For example, in the BXD population there are two alleles of the *mthfd1* gene that segregate the BXD51 and BXD2 lines as distinct from the BXD73 line. Therefore, the BXD population can be used to examine polymorphisms in other genes as they relate to choline’s ability to reduce ethanol-induced cell death.

The dose of ethanol used in the present study was a relatively high one. The rationale for this is three-fold. First, as the purpose of the present study was to evaluate the efficacy of choline in reducing ethanol-induced cell death, this dose was chosen because it has been shown to more reliably cause cell death with similar dosages used by ourselves and others (Young et al., 2005; Young and Olney, 2006; Goldowitz et al., 2014; Theberge et al., 2019; Boschen et al., 2021). Second, the alcohol exposure used in the present study models binge ethanol consumption which is the most common drinking pattern in women of child-bearing age and is characterized as episodes of high ethanol consumption in a short time window (Kanny et al., 2018; Bohm et al., 2021). Finally, the high dose used in the present study is to achieve effectiveness as small animals, including mice, metabolize ethanol at a faster rate than humans (Cederbaum, 2012).

Importantly, there are several limitations to the present study. First, the sex of the embryos remains unknown and there could be differential effects based on sex (Kwan et al., 2017). This is an important consideration and will be done in the future. Second, this study examined the acute effects of choline on mitigating ethanol-induced cell death. It will be important to determine if the effects of choline translate into long-term amelioration of ethanol-related cell loss. Third, only two doses of choline were examined and it is possible that additional differences would be detected at either higher or lower doses of choline. The current data suggest that a threshold effect was achieved at 100 mg/kg in all strains, but it would critical to know if genetics influences the minimal choline level needed, given variation in choline intake and need among clinical populations. Fourth, there could be differences in bioavailability of choline to the embryos and whether or not alcohol impacts this bioavailability. Currently, this interesting phenomenon has not been examined, but will be evaluated in future studies.

## Conclusion

In the present study, we confirm that choline is effective in ameliorating ethanol-induced cell death in the developing neural tube but further demonstrate that there are strain differences in these effects. Moreover, the differential strain effects are most pronounced at the high dose suggesting that the lowest effective dose would likely be the most beneficial and the least likely to have untoward other effects. Currently, the mechanism behind either choline’s protective effects or the molecular pathways that underlie the genetic differences are unknown. However, because the liver is an essential organ in metabolizing choline and is the first organ to encounter choline after absorption, the potential for differences in liver metabolism of choline across strains exists and this may prove to be important for understanding genetic differences in humans (Smith et al., 2021). Future research will be needed to address this issue as well as to gain a better understanding of how choline protects against ethanol’s teratogenic actions.

## Data availability statement

The raw data supporting the conclusions of this article will be made available by the authors, without undue reservation.

## Ethics statement

The animal study was approved by Institutional Animal Care and Use Committee at the University of Tennessee Health Science Centre. The study was conducted in accordance with the local legislation and institutional requirements.

## Author contributions

KH, JT, and DG contributed to conception and design of the study. KH, DG, and FX contributed to the generation of all data in the manuscript and analyses of the data. KH, FX, and DG wrote the first draft of the manuscript. All authors contributed to the article and approved the submitted version.

## Funding

This research was funded by National Institute on Alcohol Abuse and Alcoholism R01AA023508 (KH, DG), University of Tennessee Health Science Center CORNET Award (KH), and the BC Children’s Hospital Research Institute (DG).

## Conflict of interest

The authors declare that the research was conducted in the absence of any commercial or financial relationships that could be construed as a potential conflict of interest.

## Publisher’s note

All claims expressed in this article are solely those of the authors and do not necessarily represent those of their affiliated organizations, or those of the publisher, the editors and the reviewers. Any product that may be evaluated in this article, or claim that may be made by its manufacturer, is not guaranteed or endorsed by the publisher.

## Supplementary material

SUPPLEMENTARY FIGURE S1 Illustration of the regions of interest for quantification of cell death in E9.5 embryos. To the left is shown a schematic of an E9.5 embryo depicting an idealized plane of section that passes through the two regions of the brain that were analyzed: the forebrain at the level of the optic vesicle (OpV) and the brainstem at the level of the otic vesicle (OtV). To the right is shown an actual TUNEL-stained, methyl green counterstained section from an E9.5 embryo to illustrate the anatomical regions that were analyzed for cell death following the various treatments used in this study. The regions of interest analyzed are outlined in black dashed lines. The entirety of the outlined regions of interest were analyzed in appropriate sections. A = anterior, P = posterior, V = ventral, D = dorsal.

SUPPLEMENTARY FIGURE S2 Main effects of choline dose on ethanol-induced cell death irrespective of strain. Graphs represent the amount of cell death in ethanol-treated embryos that also received choline treatment with either 0 mg/kg, a moderate (100 mg/kg), or high (250 mg/kg) dose. At each dose, all the values across the 4 strains are shown in each bar. Statistical comparisons among the three ethanol-treated groups are shown in the graphs. **(A)** Main effects of choline dose in the brainstem. There is a significant effect of both the 100 mg/kg and 250 mg/kg doses on cell death compared to the 0 mg/kg dose. The two doses were equally effective at reducing cell death. **(B)** Main effects of choline dose in the forebrain. There is a significant effect of both the 100 mg/kg and 250 mg/kg dose on cell death compared to the 0 mg/kg dose. The two doses were equally effective at reducing cell death. Error bars indicate standard error of the mean (SEM). **p* < 0.05, ***p* < 0.01, ****p* < 0.001.

SUPPLEMENTARY FIGURE S3 Main effects of strain on ethanol-induced cell death irrespective of choline dose. Graphs represent the amount of cell death in ethanol-treated embryos that also received choline treatment with either 0 mg/kg, a moderate (100 mg/kg), or high (250 mg/kg) dose in each of the 4 strains: B6, BXD51, BXD2, and BXD73. For each strain, all the values across the 3 choline doses are shown in each bar. Statistical comparisons among the four strains are shown in the graphs. **(A)** Main effects of strain in the brainstem. There is a significant difference in ethanol-induced cell death between the B6 strain and BXD2 strain, between the B6 strain and BXD73 strain, between the BXD51 strain and BXD2 strain, and between the BXD51 strain and BXD73 strain. **(B)** Main effects of strain in the forebrain. There is a significant difference in ethanol-induced cell death between the B6 strain and BXD2 strain. Error bars indicate standard error of the mean (SEM). **p* < 0.05, ***p* < 0.01, ****p* < 0.001.
