# Supplementary figures and images for: The ameliorative effects of choline on ethanol-induced cell death in the neural tube of susceptible BXD strains of mice
Source: Front Neurosci. 2023 Sep 18;17:1203597. doi: 10.3389/fnins.2023.1203597 (PMC10543688; doi:10.3389/fnins.2023.1203597)

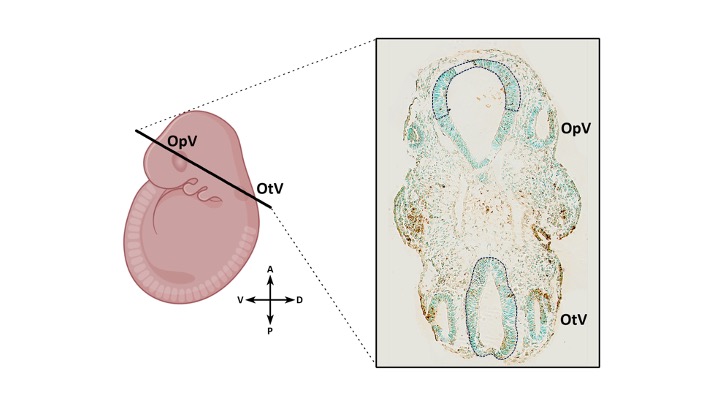

Supplement: SUPPLEMENTARY FIGURE S1 — Illustration of the regions of interest for quantification of cell death in E9.5 embryos. To the left is shown a schematic of an E9.5 embryo depicting an idealized plane of section that passes through the two regions of the brain that were analyzed: the forebrain at the level of the optic vesicle (OpV) and the brainstem at the level of the otic vesicle (OtV). To the right is shown an actual TUNEL-stained, methyl green counterstained section from an E9.5 embryo to illustrate the anatomical regions that were analyzed for cell death following the various treatments used in this study. The regions of interest analyzed are outlined in black dashed lines. The entirety of the outlined regions of interest were analyzed in appropriate sections. A = anterior, P = posterior, V = ventral, D = dorsal. [file Image_1.JPEG]

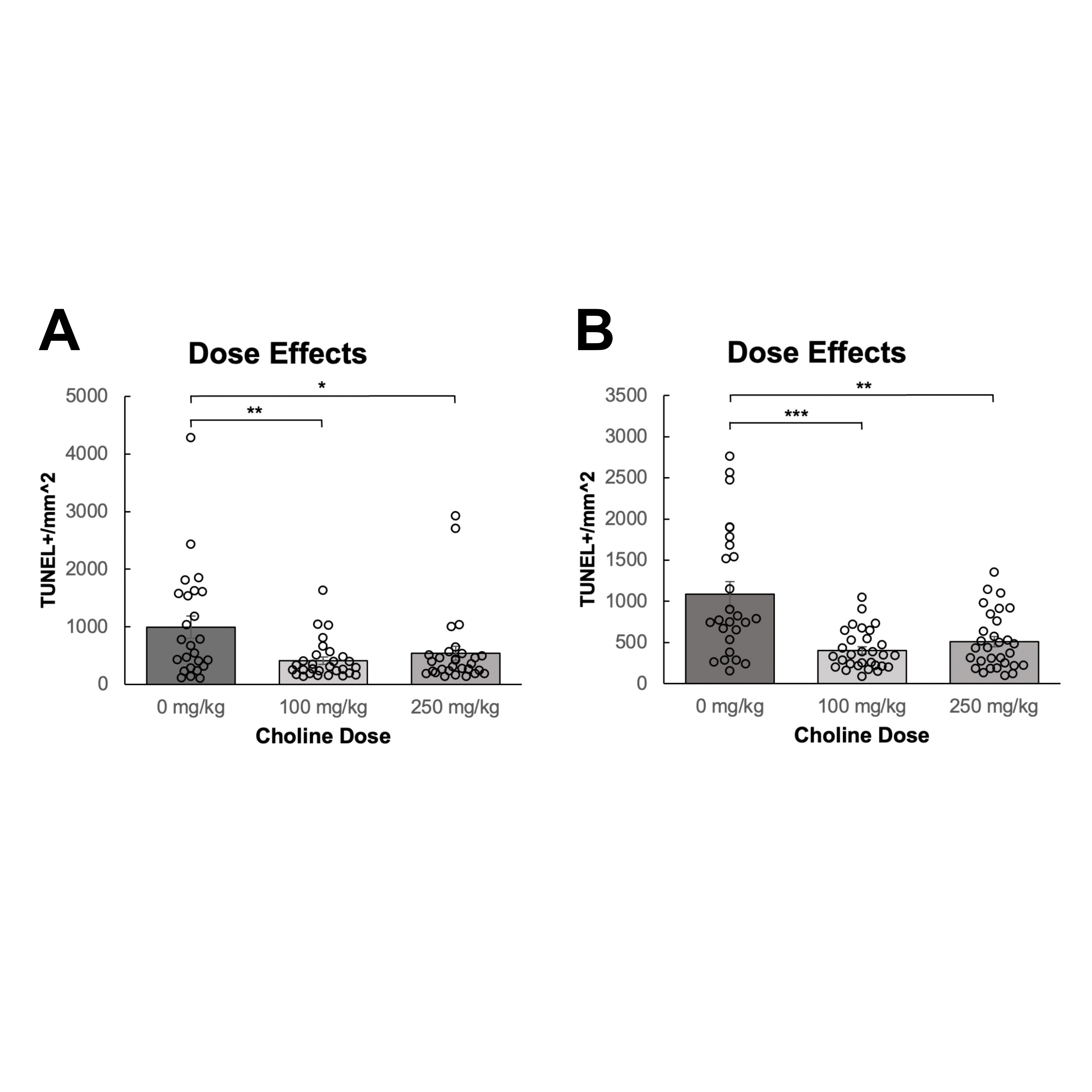

Supplement: SUPPLEMENTARY FIGURE S2 — Main effects of choline dose on ethanol-induced cell death irrespective of strain. Graphs represent the amount of cell death in ethanol-treated embryos that also received choline treatment with either 0 mg/kg, a moderate (100 mg/kg), or high (250 mg/kg) dose. At each dose, all the values across the 4 strains are shown in each bar. Statistical comparisons among the three ethanol-treated groups are shown in the graphs. (A) Main effects of choline dose in the brainstem. There is a significant effect of both the 100 mg/kg and 250 mg/kg doses on cell death compared to the 0 mg/kg dose. The two doses were equally effective at reducing cell death. (B) Main effects of choline dose in the forebrain. There is a significant effect of both the 100 mg/kg and 250 mg/kg dose on cell death compared to the 0 mg/kg dose. The two doses were equally effective at reducing cell death. Error bars indicate standard error of the mean (SEM). *p < 0.05, **p < 0.01, ***p < 0.001. [file Image_2.JPEG]

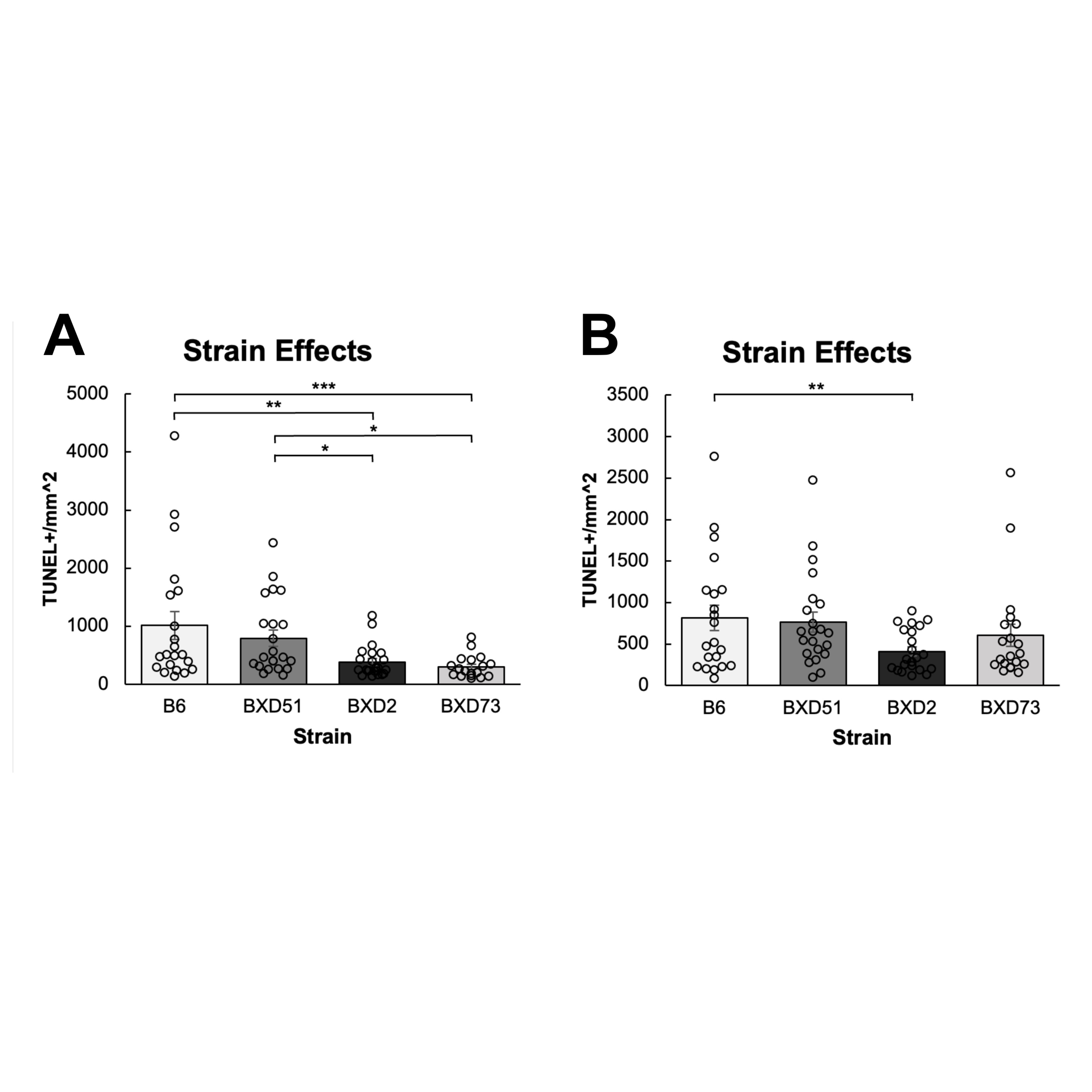

Supplement: SUPPLEMENTARY FIGURE S3 — Main effects of strain on ethanol-induced cell death irrespective of choline dose. Graphs represent the amount of cell death in ethanol-treated embryos that also received choline treatment with either 0 mg/kg, a moderate (100 mg/kg), or high (250 mg/kg) dose in each of the 4 strains: B6, BXD51, BXD2, and BXD73. For each strain, all the values across the 3 choline doses are shown in each bar. Statistical comparisons among the four strains are shown in the graphs. (A) Main effects of strain in the brainstem. There is a significant difference in ethanol-induced cell death between the B6 strain and BXD2 strain, between the B6 strain and BXD73 strain, between the BXD51 strain and BXD2 strain, and between the BXD51 strain and BXD73 strain. (B) Main effects of strain in the forebrain. There is a significant difference in ethanol-induced cell death between the B6 strain and BXD2 strain. Error bars indicate standard error of the mean (SEM). *p < 0.05, **p < 0.01, ***p < 0.001. [file Image_3.JPEG]
